# Chlorpromazine-induced perturbations of bile acids and free fatty acids in cholestatic liver injury prevented by the Chinese herbal compound Yin-Chen-Hao-Tang

**DOI:** 10.1186/s12906-015-0627-2

**Published:** 2015-04-16

**Authors:** Qiaoling Yang, Fan Yang, Xiaowen Tang, Lili Ding, Ying Xu, Yinhua Xiong, Zhengtao Wang, Li Yang

**Affiliations:** The Ministry of Education Key Laboratory for Standardization of Chinese Medicines and the State Administration of TCM (SATCM) Key Laboratory for New Resources and Quality Evaluation of Chinese Medicines, Institute of Traditional Chinese Materia Medica, Shanghai University of Traditional Chinese Medicine, 201210 Shanghai, China; Center for Chinese Medical Therapy and Systems Biology, Shanghai University of Traditional Chinese Medicine, Shanghai, 201203 China; Institute of Traditional Chinese Materia Medica, Shanghai University of Traditional Chinese Medicine, 1200 Cailun Road, Shanghai, 201210 China

**Keywords:** Bile acids, Free fatty acids, Chlorpromazine, Yin-Chen-Hao-Tang (YCHT), UPLC–MS, Hepatoprotective effect

## Abstract

**Backgrounds:**

Yin-Chen-Hao-Tang (YCHT), a commonly used as a traditional chinese medicine for liver disease. Several studies indicated that YCHT may improving hepatic triglyceride metabolism and anti-apoptotic response as well as decreasing oxidative stress .However, little is known about the role of YCHT in chlorpromazine (CPZ) –induced chlolestatic liver injury. Therefore, we aimed to facilitate the understanding of the pathogenesis of cholestatic liver injury and evaluate the effect of Yin-Chen-Hao-Tang (YCHT) on chlorpromazine (CPZ)-induced cholestatic liver injury in rats based on the change of bile acids (BAs) and free fatty acids (FFAs) alone with the biochemical indicators and histological examination.

**Methods:**

We conducted an experiment on CPZ-induced cholestatic liver injury in Wistar rats with and without YCHT for nine consecutive days. Serum levels of alanine aminotransferase (ALT), aspartate aminotransferase (AST), albumin (ALB), total bilirubin (TBIL), total cholesterol (TC), triglycerides (TG), low density lipoprotein-cholesterol (LDL-C) were measured to evaluate the protective effect of YCHT against chlorpromazine (CPZ)-induced cholestatic liver injury. Histopathology of the liver tissue showed that pathological injuries were relieved after YCHT pretreatment. In addition, ultra-performance lipid chromatography coupled with quadrupole mass spectrometry (UPLC-MS) and gas chromatography coupled with mass spectrometry (GC-MS) was applied to determine the content of bile acids, free fatty acids, respectively.

**Results:**

Obtained data showed that YCHT attenuated the effect of CPZ-induced cholestatic liver injury, which was manifested by the serum biochemical parameters and histopathology of the liver tissue. YCHT regulated the lipid levels as indicated by the reversed serum levels of TC, TG, and LDL-C. YCHT also regulated the disorder of BA and FFA metabolism by CPZ induction.

**Conclusions:**

Results indicated that YCHT exerted a protective effect on CPZ-induced cholestasis liver injury. The variance of BA and FFA concentrations can be used to evaluate the cholestatic liver injury caused by CPZ and the hepatoprotective effect of YCHT.

## Background

Cholestasis is a prevalent form of chronic liver disease characterized as a consequence of disturbed hepatocellular secretion of bile, impaired bile formation, and slow bile flow [1]. Chlorpromazine (CPZ), a member of the largest class of first-generation phenothiazine anti-psychotic drugs, is a primary drug in psychiatric treatment [2]. The hepatoxicity of CPZ should not be ignored during its therapeutic use [3]. CPZ-induced hepatotoxicity may be associated with the mechanism involving sustained activation of JNK, which leads to inflammation [4,5]. In addition, CPZ can induce cholestasis by inhibiting bile flow in vivo [6]. Previous studies on CPZ-induced intrahepatic cholestasis in vitro demonstrated that the mechanismis associated with the alteration of bile acid (BA) transport receptors and oxidative stress by altering mitochondrial membrane potential and the pericanalicular distribution of F-actin [7]. Considerable amount of evidence indicates that CPZ can be used as an excellent model of drug-induced liver injury and is usually administered to mimic drug-induced cholestasis [8-10]. However, the diagnosis and assessment of the initial toxic effects of CPZ are limited and do not accurately predict cholestasis.

The detergent character of BAs exerts an important role in regulating liver metabolism [11]. Cholestasis is an impairment or cessation of bile flow. Cholestasis leads to hepatic and systemic accumulation of potentially toxic biliary compounds, such as BAs and bilirubin, resulting in oxidative stress, apoptosis, and subsequent damage to the liver parenchyma [12]. Several studies report that the disruption of BA homeostasis is closely related to hepatic dysfunction [13-16] and intestinal ailments [17,18]. Free fatty acid (FFA) is an energy provider that plays an important role in control energy metabolism and glucose metabolism. However, FFAs can lead to cell injury and apoptosis and are key mediators of lipotoxicity within hepatocytes [19,20]. Studies indicate that abnormal FFA metabolism is associated with liver disease [21,22]. Therefore, maintaining the metabolism of BA and FFA is important for liver metabolism function. In our previous study, the validated ultra-performance lipid chromatography coupled with quadrupole mass spectrometry (UPLC–MS) method based on BA and gas chromatography coupled with mass spectrometry (GC–MS) based on FFA were applied to evaluate the carbon tetrachloride, α-naphthylisothiocyanate (ANIT) and acetaminophen-induced liver injury in rats [23,24].

YCHT is a famous and classic Chinese herbal compound that consists of three medicinal materials, namely, *Artemisia capillaris* Thunb (Tarragon), *Gardenia jasminoides* Ellis (Gardenia), and *Rheum officinale* Baill (Rhubarb). YCHT is recorded in “Shang Han Lun” and has been used to treat jaundice for more than a thousand years. YCHT is considered as a hepatoprotective agent by improving hepatic triglyceride metabolism and anti-apoptotic response as well as decreasing oxidative stress [25-30]. Related proteomics data suggest that the therapeutic effects of YCHT may be associated with the regulation of lipid biosynthesis [31]. Limited data are available about the efficacy of YCHT on CPZ-induced cholestasis and its corresponding mechanism.

This study aimed to evaluate the protective effect of YCHT on CPZ-induced cholestatic liver injury based on the variations of endogenous metabolites and provide insights into the role of BAs and FFAs in the progression of the pathological changes.

## Methods

### Chemicals and reagents

Rhubarb was provided by Shanghai Hutchison Pharmaceuticals (batch number: 121012; Shanghai, China). Gardenia and Tarragon were purchased from Shanghai Cambridge Traditional Chinese Medicine decoction pieces company (batch number: 081226; Shanghai, China) and Bozhou (batch number: 20100708; Anhui, China) medicine market, respectively. They were authenticated as *Rheum officinale* Baill, *Gardenia jasminoides* Ellis, *Artemisia capillaris* Thunb by Dr. LiHong Wu (Professor, Instituent of Chinese Materia Medica, Shanghai University of Tradational Chinese Medicine). The voucher specimens (dh-121012, ych-20100708, zz-081226) were deposited in the Herbarium of Instituent of Chinese Materia Medica, Shanghai University of Traditional Chinese Medicine. CPZ hydrochloride injection was purchased from Shanghai Harvest Pharmaceutical Co., Ltd. α-muricholic acid (α-MCA), β-muricholic acid (β-MCA), ω-muricholic acid (ω-MCA), Cholic acid (CA), deoxycholic acid (DCA), chenocholic acid (CDCA), lithocholic acid, ursodeoxycholic acid, hyodesoxycholic acid, glycocholic acid (GCA), taurocholic acid (TCA), glycodeoxycholic acid (GDCA), taurodeoxycholic acid (TDCA), glycochenodeoxycholic acid (GCDCA), taurochenodeoxycholic acid (TCDCA), glycoursodeoxycholic acid, taurohyodesoxycholic acid (THDCA), glycolithocholic acid (GLCA), taurohyodesoxycholic acid (TLCA), lauric acid (C12:0), tetradecanoic acid (C14:0), palnitic acid (C16:0), heptadecanoic acid (C17:0), stearic acid (C18:0), arachidic acid (C20:0), docosanoic acid (C22:0), lignoceric acid (C24:0), palmitoleic acid (C16:1n7), oleic acid (C18:1n9), vaccenic acid (C18:1n7), linoleic acid (C18:2n6), γ-linolenic acid (C18:3n6), linolenic acid (C18:3n3), eicosatrienoic acid (C20:3n6), arachidonic acid (C20:4n6), eicosapentaenoic acid (C20:5n3), and docosahexaenoic acid (C22:6n3) were purchased from Sigma-Aldrich. Their purities were above 98%. Acetonitrile, methanol, formic acid, and ammonium acetate (HPLC grade) were purchased from Fisher Scientific (Nepean, Ontario, Canada). De-ionized water was prepared by Milli-Q system (Millipore, Bedford, MA). The other solvents were of analytical grade and obtained from Shanghai Chemical Factory (Shanghai, China).

### Preparation of YCHT and chemical analysis by UPLC-QTOF/MS/MS

YCHT was extracted as follows. Crude drug materials of *Rhubarb* (30 g), *Gardenia* (45 g), and *Artemisia capillaries* (90 g) were decocted three times in boiling water (3000 mL) for 1.5 h each time. The decoctions were filtered, combined, and concentrated to the volume of 300 mL. Liquid chromatography/electrospray ionization time-of-flight mass spectrometry (LC/ESI-TOF MS) was adopted to validate the chemical composition of the aqueous extract of YCHT. Samples were separated on the Waters ACQUITY BEH C18 column (100*2.1 mm, 1.7 μm) with the column temperature maintained at 45°C. The mobile phase consisted of 0.1% formic acid in 5 mM ammonium acetate aqueous solution (A) and methanol (B) at a flow rate of 0.3 mL/min. The elution gradient was performed as follows. During the first 1 min, the eluent composition was set at 95% A and 5% B, which was linearly changed to 75% A and 25% B in 4 min, and then the proportion of B was increased to 50% in the next 20 min. The proportion of B was linearly increased by 95% in the next 6 min. The sample injection volume was 5 μL. Mass spectrometry was performed on the Waters SYNAPT QTOF/MS (Waters Corp.). The mass range was set at m/z 100 Da to 1200 Da. The MS/MS experiments were performed at variable collision energy (20 eV to 30 eV). The data were processed using MassLynx 4.1 software (Waters Corp.).

### Ethics statement

The Guide for the Care and Use of Laboratory Animals was strictly complied, and the animal experiment protocols were approved by the Institutional Animal Committee of Shanghai University of Traditional Chinese Medicine [Permit number: SCXK (Hu) 2012–0002]. All surgical procedures were performed under ether, and all efforts were made to reduce animal suffering.

### Animal administration and sample collection

Male Wistar rats (220 ± 20 g, 6 weeks to 8 weeks of age) were obtained from the laboratory animal center of Shanghai University of Traditional Chinese Medicine (SHUTCM, Shanghai). The animals were maintained on a 12/12 h light–dark cycle (lights on at 7:00 am) with regulated temperature and humidity. During the whole experimental process, rats were fed with certified standard rat chow and tap water ad libitum. All rats were allowed to acclimatize for 7 days before experimentation and randomly divided into three groups (eight rats for each group). Group 1 served as non-treated controls, and group 2 served as CPZ-treated model group. The rats of group 3 were intragastrically given with YCHT, which was suspended in distilled water at doses of 8 g/kg (10 mL/kg, B.W.) every 24 h for nine consecutive days. At 12 h after administration of the seventh dose, the rats of groups 2 and 3 received CPZ by intraperitoneal injection at a dose of 75 mg/kg (3.6 mL/kg, B.W.), which is well documented to induce liver injury and cholestasis. Meanwhile, group 1 received intragastrical treatment of physiological saline in an equal volume as for groups 2 and 3 (5 mL/kg B.W.). At the end of the study, all rats were euthanized by CO_2_ inhalation in a 12-h fasting state. Retro-orbital blood samples were collected into tubes at 48 h after the last treatment and then immediately centrifuged at 4°C for 10 min (3000 g) to separate the serum. The resulting serum samples were stored at −80°C until analysis. Each liver sample was isolated and stored at −80°C for further analysis, except for the central part of the right large lobe, which was used for histological examination.

### Biochemical determination and histological examination

The collected blood samples were placed at room temperature for 4 h and centrifuged at 13000 g for 10 min at 4°C to obtain serum. The serum contents of ALT, AST, ALB, TBIL, TC, TG, and LDL-C were determined using a commercially available clinical test kit and a chemistry analyzer system (HITACHI 7080; Japan). The liver samples obtained from the central part of the right large lobe were fixed with 10% formalin in PBS for 24 h and then washed with tap water, dehydrated in alcohol, and embedded in paraffin. The 4 μm-thick sections were obtained, deparaffinized, dehydrated in ethanol (50% to 100%), and cleared with xylene. Each slide was stained with hematoxylin and eosin, and then histological assessment was performed by Shanghai Showbio Biotech, Inc.

### Quantitative determination of BAs

The BA quantification method [23] was conducted with modification. The BA mix reference standards were prepared by dissolving each BA in methanol. BA was extracted in serum in serum as follows. In brief, 300 μL of methanol was added to 100 μL of serum, and the mixture was vortexed for 2 min and centrifuged (20000 g, 4°C) for 10 min. The supernatant was separated and evaporated to dryness, and the residue was stored at −20°C and reconstituted in 100 μL of methanol–water (55:45; containing a mixture of 5 mM ammonium acetate and 0.1% formic acid) before analysis. The sample solution was centrifuged at 20000 g for 10 min at 4°C, and a 5 μL of aliquot was injected for UPLC–MS analysis.

BAs were determined using the Waters ACQUITY ultra-performance lipid chromatograph system (Waters, MA, USA) equipped with the Acquity UPLC BEH C18 (1.7 μm, 2.1*100 mm, Waters) column with a temperature of 45°C. The mobile phase consisted of 0.1% formic acid in 5 mM ammonium acetate aqueous solution (A) and methanol (B) at a flow rate of 0.3 mL/min. The elution gradient was performed as follows. During the first 1 min, eluent composition was set at 55% A and 45% B, which was linearly changed to 62% A and 38% B in 2.6 min, and then the proportion of B was increased to 80% in the next 8.8 min. The sample injection volume was 5 μL.

MS analysis was performed using ZQ 2000 quadrupole spectrometry equipped with an ESI probe operated with Selective Ion Monitoring (SIM) in the negative-ion mode (Waters, MA, USA). The capillary and cone voltages were set at 3.0 and 55 V, respectively. The source temperature was 120°C, and the desolvation temperature was 300°C. The desolvation gas flow was set at 700 L/h, and the cone gas flow rate was set at 50 L/h. Data were acquired and processed using MassLynx 4.1 software.

### Quantitative determination of FFAs

The quantification method [24] was conducted with modification. A mixed standard solution of fatty acid methyl esters was prepared in 5% H_2_SO_4_-CH_3_OH. The FFAs in serum were extracted. Twenty microliters of 1000 μg/mL mixed internal standard (C19:2n10 and its methyl ester) was added to 100 μL of serum. FFAs were methylated in 5% H_2_SO_4_-CH_3_OH. Lipid extraction was performed using *n*-hexane. The *n*-hexane phase was collected, evaporated to dryness in the N_2_ atmosphere, and re-dissolved by 500 μL of *n*-hexane.

Experiments were performed on a 6800 GC system (Agilent Technologies, Santa Clara, CA, USA) coupled with a 5973 mass spectrometer. The GC system was equipped with a 7683B series injector. The chromatographic separation was performed with a DB-225 MS capillary column (60 m*0.25 mm i.d., 0.25 μm film thickness, Agilent, Folsom, CA, USA). The oven gradient temperature was performed as follows. During the initial 1 min, the temperature was set at 70°C, increased to 200°C by 40°C/min in the next 20 min, changed to 230°C by a second gradient of 5°C/min, and held for 25 min. A 5973 mass spectrometer in the electron impact was operated at 70 eV on the SIM mode. The temperatures of the ion source and quadrupole were adjusted to 230 and 150°C, respectively.

### Data processing and statistical analysis

UPLC–MS and GC–MS data were acquired and processed using MassLynx 4.1 and enhanced MSD ChemStation software (Agilent Technologies, Inc., USA), respectively. Statistics was analyzed with one-way ANOVA and the least significant difference test (SPSS 18.0 software, Inc., Chicago, USA). The difference was considered statistically significant when *p* ≤ 0.05, very significant when *p* ≤ 0.01, and highly significant when *p* ≤ 0.001. Multivariate statistical analysis was conducted by SIMCA-P 11.5 (Umetrics, Umea, Sweden).

## Results

### Chemical analysis of YCHT

**Figure 1:**
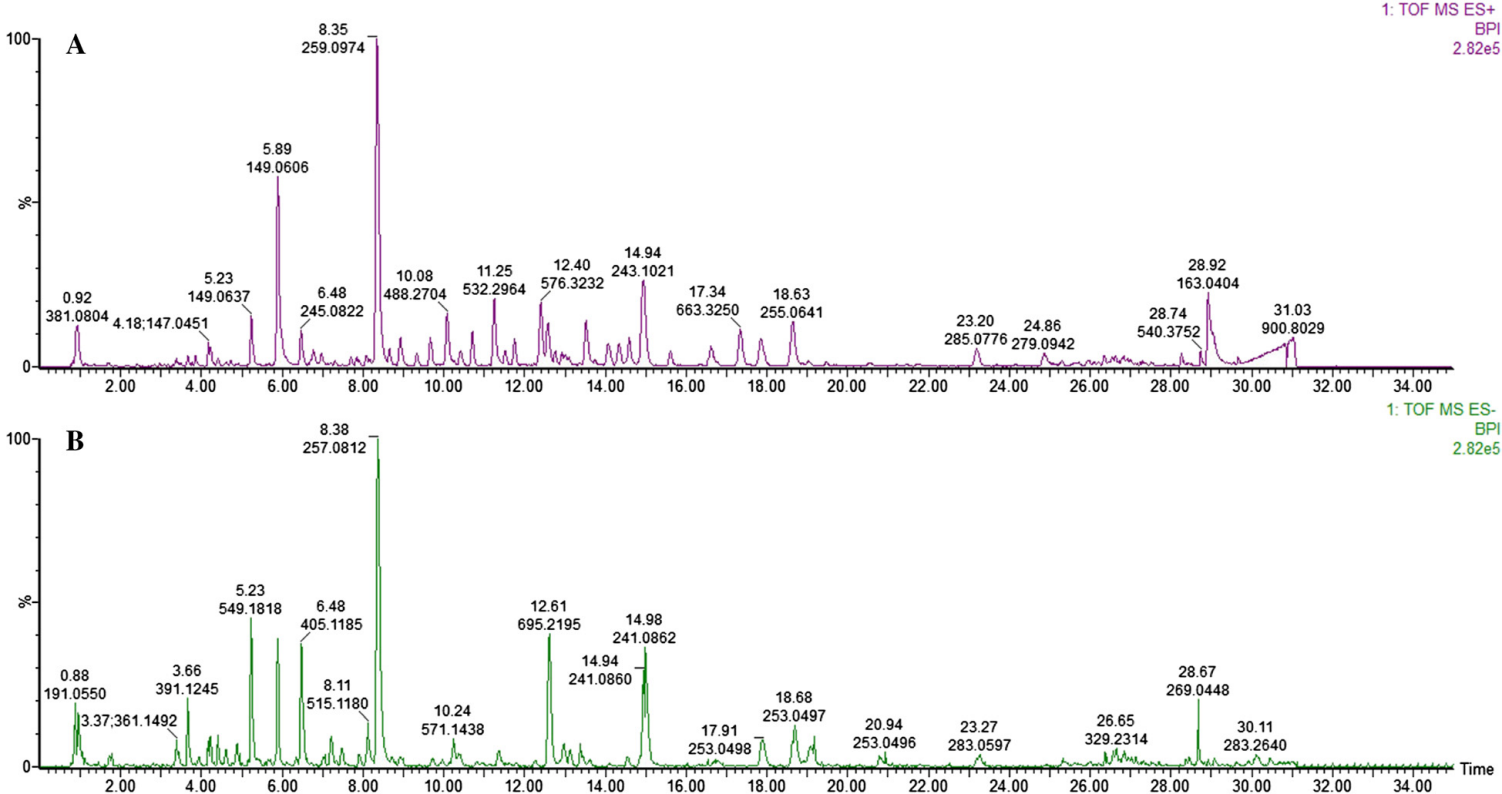
Chromatogram of the aqueous extract of YCHT by UPLC-QTOF/MS/MS on positive-ion polarity mode **(A)** and negative-ion polarity mode **(B)**.

The aqueous extract of YCHT was analyzed by Waters SYNAPT G2 QTOF/MS. The UPLC-QTOF/MS chromatogram is shown in Figure 1. The separated compounds were clarified by comparing the Rt values and the MS characteristics in both positive- and negative-ion polarity modes (see Table 1).

**Table 1:** **Main compounds in the aqueous extract of YCHT by UPLC-QTOF/MS/MS**

**Peaks**	**Retention time (min)**	**M/Z**	**Identified compounds**
1	0.84	181.0715[M-H]^−^	Mannitol
2	1.79	169.0135[M-H]^−^	Gallic acid
3	3.63	373.1123[M-H]^−^	Geniposidic acid
4	3.66	391.1245[M-H]^−^	Gardenoside
5	3.93	137.023[M-H]^−^	3,4-dihydroxybenzaldehyde
6	4.34	353.0870[M-H]^−^	Chlorogenic acid
7	4.35	153.0192[M-H]^−^	3,4-dihydroxybenzoic acid
8	4.83	515.1190[M-H]^−^	1,3-dicaffeoylquinic acid
9	5.23	549.1811[M-H]^−^	Genipin-1-β-D-gentiobioside
10	5.66	135.0446[M-H]^−^	4-hydroxyacetophenone
11	5.88	387.1285[M-H]^−^	Geniposide
12	5.88	225.0766[M-H]^−^	Genipin
13	7.21	477.1404[M-H]^−^	Isolindleyin
14	7.47	183.102[M-H]^−^	Jasminodiol
15	7.63	463.0869[M-H]^−^	Isoquercitrin
16	8.11	515.1196[M-H]^−^	3,5-dicaffeoylquinic acid
17	8.31	207.0659[M + H]^+^	7-dimethylesculetin
18	8.32	419.1357[M-H]^−^	Poniticin
19	8.35	445.0760[M-H]^−^	Rhein-1-O-β-D-glucopyranoside
20	10.37	515.1189[M-H]^−^	4,5-dicaffeoylquinic acid
21	12.6	695.1295[M-H]^−^	6-O-trans-coumaroylgenipin-gentiobioside
22	12.97	755.2415[M-H]	6-O-trans-sinapolygenipin-gentiobioside
23	14.86	593.1865[M-H]^−^	6-O-sinapolygeniposide
24	23.27	283.0759[M-H]^−^	Physcion
25	25.35	283.0247[M-H]^−^	Rhein
26	25.47	239.0343[M-H]^−^	Alizarin
27	28.76	269.0451[M-H]^−^	Emodin
28	29.12	169.0135[M-H]^−^	Chrysophanol

### YCHT reversed the alterations of serum biochemicals in rats with CPZ-induced cholestatic liver injury

**Figure 2:**
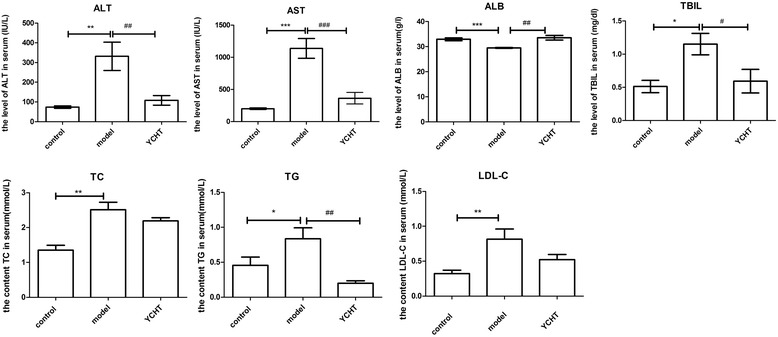
Effects of YCHT on ALT, AST, ALB, TBIL, TC, TG, and LDL-C in serum after CPZ treatment. The results are expressed as mean ± SEM. ^*^ *p* < 0.05, ^**^ *p* < 0.01, ^***^ *p* < 0.001, significantly different from the control group. ^#^ *p* < 0.05, ^##^ *p* < 0.01, ^###^ *p* < 0.001, significantly different from the model group.

Several clinical parameters in the serum were measured to monitor the toxic effects of CPZ and confirm the occurrence of cholestatic liver injury induced by CPZ in the animal model. Alone administration of CPZ induced a significant increase in serum level of ALT, AST, ALB, TBIL in rats as compared to normal control group, suggested that CPZ exposure has successfully lead to cholestatic liver injury. Other clinical parameters measured in serum were also significantly changed. TC, TG, and LDL-C were significantly increased in the model group compared with those in the control group, which indicates that CPZ exposure may affect lipid metabolism. However, the group pretreated with YCHT significantly declined the CPZ-induced elevation in the serum levels of ALT, AST, ALB, TBIL, TC, TG, and LDL-C, and concentration of TC (Figure 2 and Table 2). No adverse health effects on rats were observed during the experiment.

**Table 2:** **Effect of YCHT on the biochemical parameters of serum**

**Group**	**ALT**	**AST**	**ALB**	**TBIL**	**TC**	**TG**	**LDL-C**
	**(IU/L)**	**(IU/L)**	**(IU/L)**	**(mg/dl)**	**(mmol/L)**	**(mmol/L)**	**(mmol/L)**
control	74.17 ± 6.42	202.3311.16	32.93 ± 0.55	0.51 ± 0.09	1.35 ± 0.14	0.465 ± 0.12	0.327 ± 0.05
model	331.33 ± 71.78^**^	1138 ± 153.53^***^	29.5 ± 0.17^**^	1.15 ± 0.16	2.38 ± 0.21^**^	0.847 ± 0.16^*^	0.82 ± 0.15^**^
YCHT	108.33 ± 24.56^##^	363.83 ± 89.19^###^	33.57 ± 0.9^###^	0.59 ± 0.18	2.32 ± 0.19	0.2 ± 0.03^##^	0.52 ± 0.07

The results are expressed as mean ± SEM. ^*^ *p* < 0.05, ^**^ *p* < 0.01, ^***^ *p* < 0.001, significantly different from control group. ^#^ *p* < 0.05, ^##^ *p* < 0.01, ^###^ *p* < 0.001, significantly different from model group.

### Effect of YCHT on histological changes

**Figure 3:**
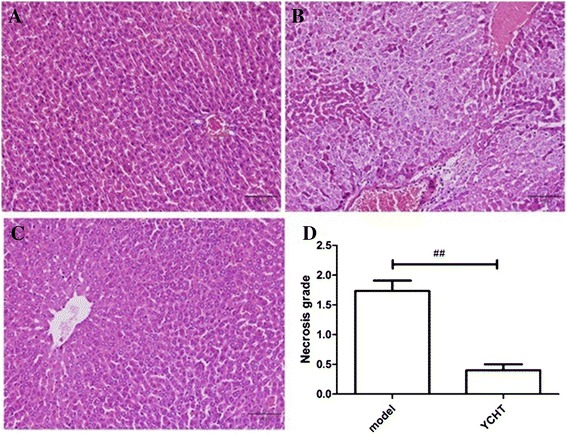
Effect of YCHT on histological changes in the control group** (A)**, model group** (B)**, YCHT group **(C)** and necrosis grade **(D)**.

Main changes, such as proliferation of bile duct, expansion of hepatic sinus, necrosis of hepatocyte, and effusion of inflammation factors, were observed in CPZ-stimulated hepatotoxicity animal models (Figure 3B). However, these changes were suppressed in the liver sections of rats pretreated with YCHT. This finding indicates mild necrosis of hepatocyte and effusion of inflammation factors as shown in Figure 3C.In addition, the necrosis of hepatocyte was confirmed by the quantitative scoring (Figure 3D).

### YCHT affects the serum BA and FFA profiles in rats with CPZ-induced cholestatic liver injury

**Figure 4:**
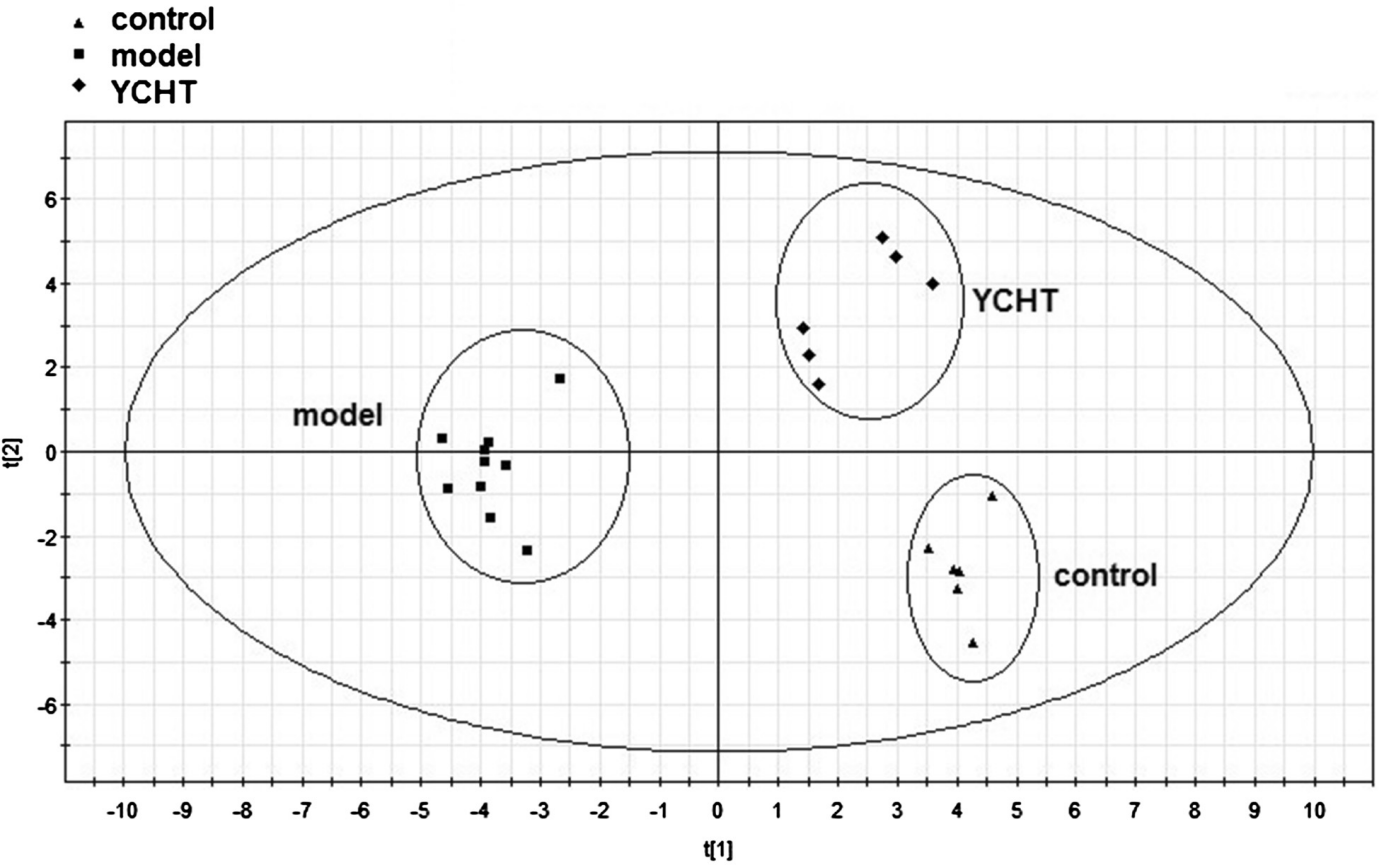
PLS-DA score plot derived from three representative control, model, and YCHT groups using serum BAs and FFAs.

The characterization and quantification of BAs and FFAs in serum are focused in the study of metabolic progress. Alterations in BA and FFA profiles are observed in nutritional diseases, metabolic disorders, obesity, cancer, and gastrointestinal diseases. Thus, the optimized reversed-phase UPLC–MS and GC–MS conditions were applied to determine the contents of individual BA and FFA in serum and further interpret the biological events. The data based on the quantitative analysis of BAs and FFAs were exported to SIMCA-P software for the multivariate statistical analysis in the form of principal component analysis and partial least squares discriminant analysis (PLS-DA). The parameters adopted to evaluate the model quality included R^2^ and Q^2^. The R^2^ values indicated the explained variation, and the Q^2^ values indicated the predictive ability. Figure 4 displays the result of PLS-DA model, which shows clusters and separations from the control, model, and YCHT groups, indicating that CPZ injection affected the metabolism of BAs and FFAs. The group pretreated with YCHT was located between the model and control groups, indicating that YCHT gradually adjusted the pathological condition to physiological condition. Combined the selected variables with VIP values larger than 1 and the significant statistical analysis, α-MCA, β-MCA, CA, DCA, TCDCA, TDCA, THDCA, GCA, HDCA,UDCA,C18:1n9, C18:2n6, and C20:5n3 were recognized as the most important parameters for elucidating the cholestasis process and evaluating the effect of YCHT on CPZ-induced cholestatic liver injury.

**Figure 5:**
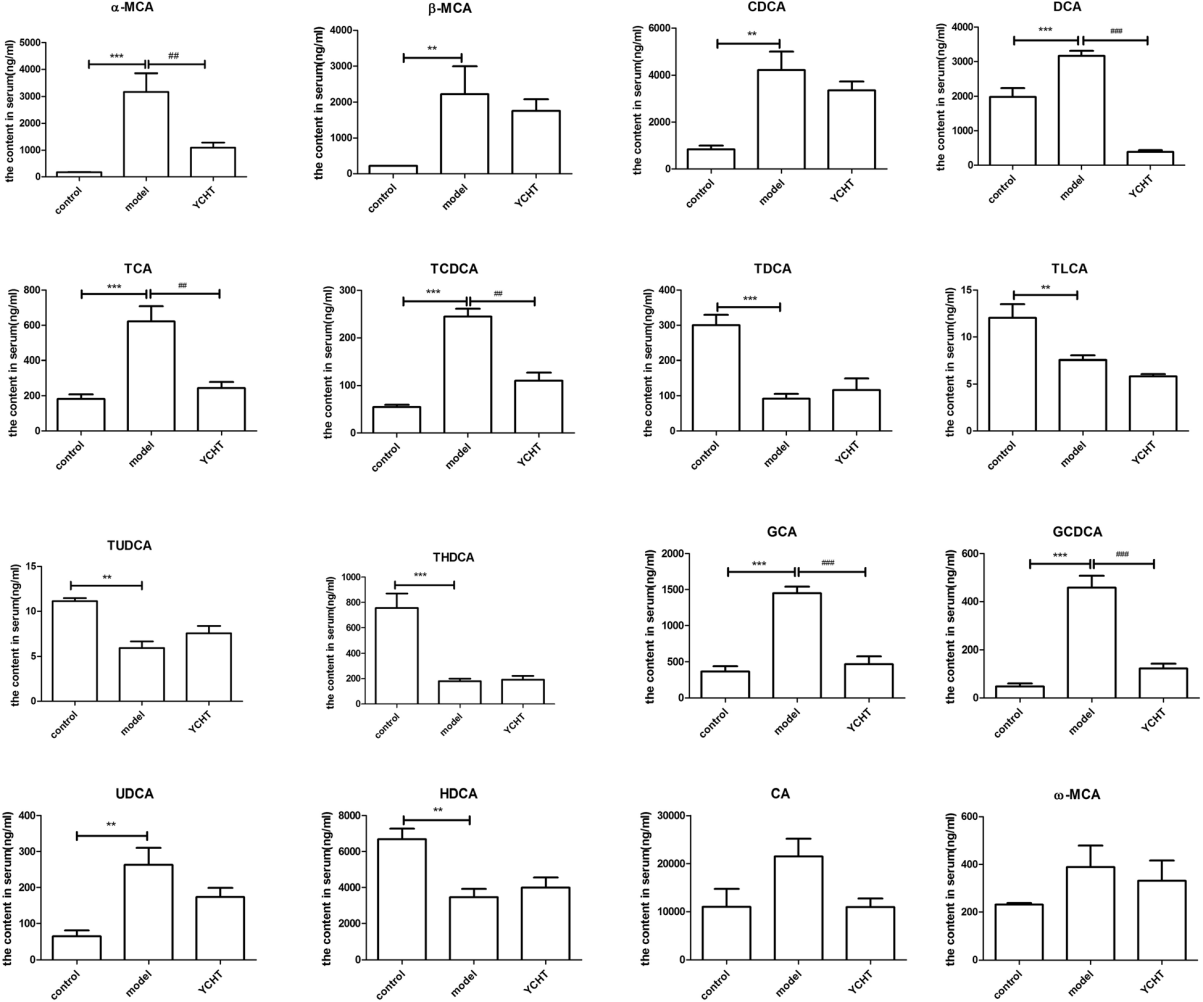
Changes in serum concentrations of BAs identified in different groups (control, model, and YCHT). The results are expressed in ng/mL as mean ± SEM. ^*^ *p* < 0.05, ^**^ *p* < 0.01, ^***^ *p* < 0.001, significantly different from the control group. ^#^ *p* < 0.05, ^##^ *p* < 0.01, ^###^ *p* < 0.001, significantly different from the model group.

BAs are recognized as regulatory molecules that are involved in major metabolic progress and show dynamic variances. In this study, the quantitative results and variation tendencies of serum BA profiling are shown in Figure 5 and Table 3, respectively. BA concentration significantly varied in the three groups. Increasing primary BA concentrations were detected in rats with CPZ-induced cholestatic liver injury. However, the serum concentration of secondary BAs decreased in rats with CPZ-induced cholestatic liver injury compared with that in the control group. Compared with the control group, increasing α-MCA, β-MCA, ω-MCA, CA, CDCA, and corresponding conjugated BAs were observed in the model group, whereas secondary BAs and corresponding conjugated BAs decreased, except for DCA. The groups pretreated with YCHT showed reversed effect.

**Table 3:** **Effect of YCHT on bile acids (BAs) and free fatty acids (FFAs) metabolism**

**Compound**	**Control**	**Model**	**YCHT**
CA	10959.98 ± 3777.06	21454.91 ± 3738.66	10936.3 ± 1774.1
α-MCA	175.72 ± 4.15	3171.48 ± 690.76^***^	1092.45 ± 188.1^##^
β-MCA	218.17 ± 5.07	2222.1 ± 771.15^**^	1754.07 ± 333.24
ω-MCA	231.65 ± 5.85	388.25 ± 90.3	331.23 ± 84.9
CDCA	832.91 ± 157.56	4203.03 ± 796.24^**^	3348.31 ± 377.81
DCA	1979.46 ± 262.43	3166.18 ± 144.98^***^	383.96 ± 54.89^###^
LCA	24.46 ± 2.48	14.07 ± 1.19^*^	21.44 ± 4.16
UDCA	65.251 ± 15.73	262.81 ± 47.24^**^	173.88 ± 25.20
HDCA	6679.97 ± 598.89	3464.91 ± 463.26^**^	3997.04 ± 564.22
TCA	192.81 ± 25.91	621.46 ± 85.46^***^	244.33 ± 34.33^##^
TCDCA	54.39 ± 7.79	244.88 ± 5.17^***^	110.19 ± 17.09^##^
TDCA	299.94 ± 29.46	91.63 ± 13.68^***^	115.99 ± 39.34
TLCA	12.03 ± 1.45	7.53 ± 0.49^**^	5.81 ± 0.24
TUDCA	11.12 ± 0.34	5.94 ± 0.70^***^	7.55 ± 0.80
THDCA	757.34 ± 112.71	180.02 ± 19.41^***^	192.5 ± 28.36
GCA	364.43 ± 72.98	1449.35 ± 89.25^***^	466.99 ± 110.79 ^###^
GCDCA	47.42 ± 12.70	458.01 ± 49.91^***^	122.86 ± 12.03^###^
GDCA	644.59 ± 147.35	21.98 ± 3.8^***^	196.62 ± 36.89
GLCA	17.93 ± 1.56	13.55 ± 0.76^**^	13.62 ± 0.71
C 12:0	2.81 ± 0.021	2.60 ± 0.048	2.47 ± 0.03
C 14:0	2.78 ± 0.12	2.64 ± 0.08	2.23 ± 0.08
C 16:0	54.81 ± 2.12	59.43 ± 2.83	42.74 ± 2.8
C16:1n7	1.77 ± 0.05	1.88 ± 0.08	1.53 ± 0.06
C18:0	35.84 ± 1.47	37.49 ± 2.41	29.23 ± 2.33
C18:1n9	5.867 ± 0.11	8.58 ± 0.75^**^	4.30 ± 0.34^###^
C18:1n7	1.90 ± 0.89	2.01 ± 1.68	1.75 ± 0.73
C18;2n6	12.29 ± 0.3	18.2 ± 0.04^**^	8.69 ± 0.03^###^
C18:3n3	1.45 ± 0.03	1.546 ± 0.03	1.29 ± 0.03
C20:0	1.56 ± 0.05	1.532 ± 0.03	1.45 ± 0.01
C20:3n6	1.48 ± 0.03	1.65 ± 0.03^**^	1.53 ± 0.01^#^
C20:4n6	10.60 ± 0.50	14.74 ± 0.61^***^	10.92 ± 0.77^###^
C20:5n3	2.33 ± 0.05	2.58 ± 0.16^*^	2.08 ± 0.03^###^
C22:6n3	4.91 ± 0.21	7.73 ± 0.67^***^	4.95 ± 0.37^###^

The results are expressed in ng/mL as mean ± SEM. ^*^ *p* < 0.05, ^**^ *p* < 0.01, ^***^ *p* < 0.001, significantly different from control group. ^#^ *p* < 0.05, ^##^ *p* < 0.01, ^###^ *p* < 0.001, significantly different from model group.

**Figure 6:**
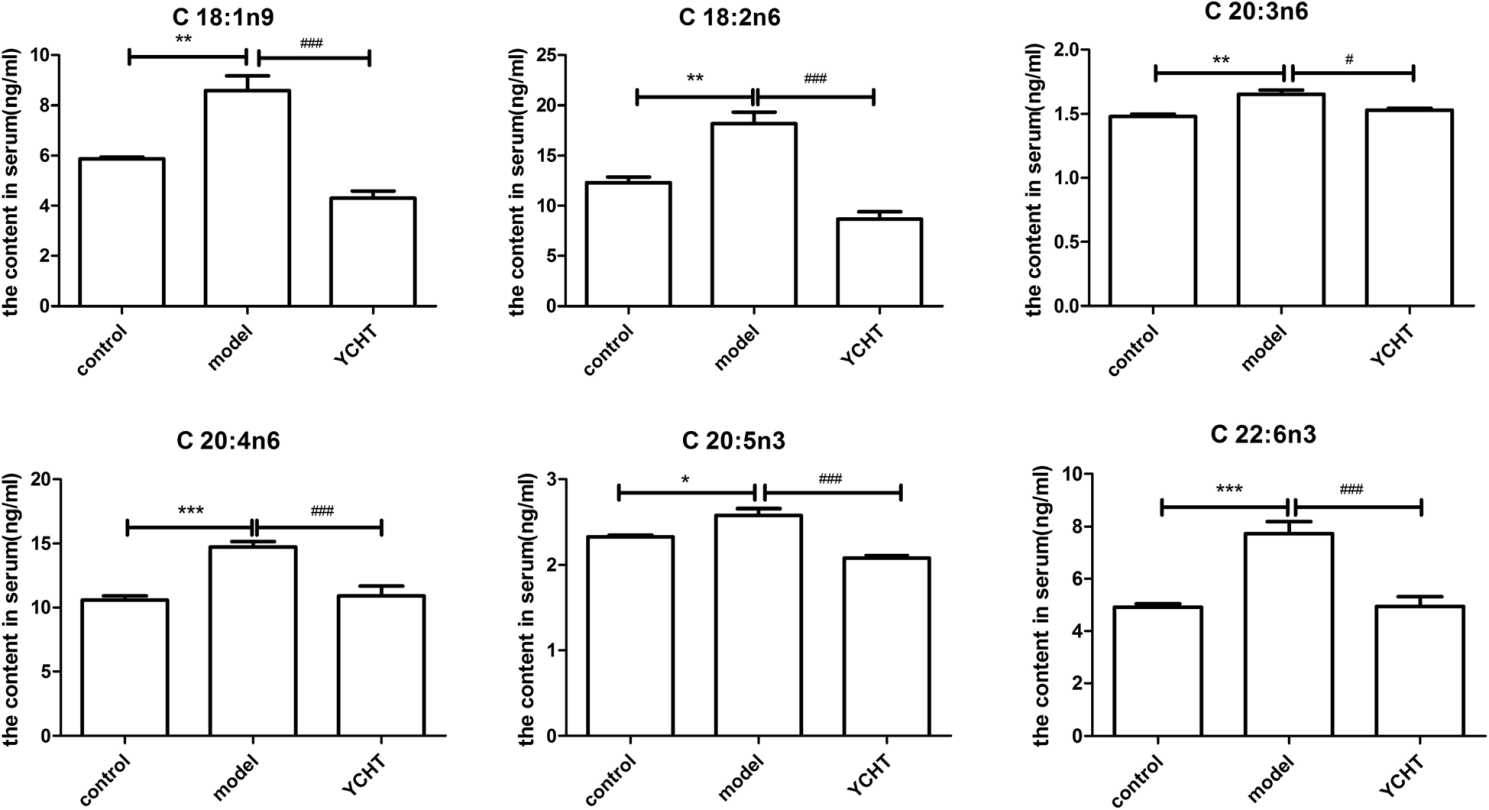
Changes in the serum concentrations of FFAs identified in different groups (control, model, and YCHT). The results are expressed in ng/mL as mean ± SEM. ^*^ *p* < 0.05, ^**^ *p* < 0.01, ^***^ *p* < 0.001, significantly different from the control group. ^#^ *p* < 0.05, ^##^ *p* < 0.01, ^###^ *p* < 0.001, significantly different from the model group.

In addition, the FFA concentrations in the serum of the control, model, and treated groups were quantified by the optimal GC–MS conditions described in this study. These FFA concentrations in the three groups were compared by one-way ANOVA with LSD post hoc analysis. Despite large variations among individuals, dynamic variance was observed for several FFAs. The variations mainly involved C18:1n9, C18:2n6, C18:3n3, C20:4n6, C20:5n3, and C22:6n3, as labeled in Figure 6 and summarized in Table 3.

## Discussion

Cholestasis is a common chronic liver disease with variable frequency that confers risks of progression to severe disease and development of end-stage liver disease or specific disease variants. Cholestasis is characterized as a consequence of the disruption of BA homeostasis, the impairment of liver antioxidant defense system or mitochondrial dysfunction. Both hepatocellular functional defects and obstructive lesions of the small bile duct lead to cholestatic liver injury. Cholestasis causes the accumulation of BAs in liver and limit the elimination of BAs in hepatocytes. Accumulated BAs in hepatocytes result in oxidative stress, promote hepatocyte necrosis, and liver apoptosis. Meanwhile, the mechanism of cholestasis is often associated with hepatocellular transporter expression [32-36]. Previous work reports that the mechanism of CPZ-induced cholestatic liver injury is associated with inhibition of BSEP and MDR3 transcript levels [7]. In this study, the data showed that cholestatic liver injury induced by CPZ perturbed BA homeostasis, manifested by the elevated hydrophobic BAs (α, β, ω-MCA, DCA, CDCA, and CA) in serum. The retention of hydrophobic BAs can result in mitochondrial dysfunction by generating ROS, which in turn causes liver injury [37]. However, the group pretreated with YCHT restrained the variance of serum levels of hydrophobic BAs (α, β, ω-MCA, DCA, CDCA, and CA).

BAs are substrates of BA coenzyme A synthase and BA amino acid transferase by conjugating to amino acids (glycine and taurine) that make them more hydrophilic at acidic pH. These substrates are subsequently impermeable to cell membrane and minimize passive absorption. Similar to drug conjugation, the impermeable properties of the conjugated BAs lead to efficient transportation and detoxification. In this study, the increased contents of serum TCA, GCA, TCDCA, and GCDCA in the model group are in accordance with previous reports [38,39]. This result may be explained as the consequence of the liver, which reacts with the adaptive response for limiting the hepatic BA overload. In addition, the elevated level of serum conjugated BAs may be caused by the alteration of transporter protein. The significant increases of serum TCA and GCA in the model group in this study were attributed to the multidrug resistance proteins (Mrp2/Mrp3) that possess high affinity for TCA and GCA. YCHT showed reverse activities on cholestatic liver injury based on the alterations of concentration of conjugated BAs. These observations indicate that YCHT protects the liver from cholestatic liver injury by reducing the size of the total BA pool (data not shown). Basing on this finding, we speculate that the mechanism of the cholestatic liver injury induced by CPZ and the protection of YCHT may be associated with the regulation of BA metabolism.

FFA, an intracellular signaling sensor of PPARα, participates in lipid metabolism, glucose metabolism, BA metabolism, and inflammation. Evidence demonstrates that the disturbance in lipid homeostasis is causally associated with the pathogenesis and progression of cholangiopathies and biliary fibrosis [21,40]. Previous report suggests that FFAs contribute to significant up-regulation of NTCP and Cyp7A1 through the induction of the FXR-SHP pathway [22]. Therefore, this study explored the indicators related to lipid metabolism and discovered that the serum levels of biochemical indicators, including TC, TG, and LDL-C, were increased in the model group. The findings also suggested that CPZ induced the disturbance of lipid metabolism. Moreover, the data based on the metabolic profiling of FFAs were reported in this study, which also indicate the disruption of lipid metabolism in the progression of cholestasis. Pretreatment with YCHT showed the reverse effect in the disruption of lipid metabolism induced by CPZ.

Given the limited availability of drugs to treat hepatobiliary diseases, more anti-cholestasis agents that are safe, effective, and well-characterized are needed. YCHT, a well-known TCM, has an anti-apoptotic property, and is considered a hepatoprotective agent and an antioxidant associated with lipid biosynthesis and peroxidant regulation [26,27,40-44]. Previous reports show that YCHT protects against liver injury with cholestasis in animals having bile duct ligation [25,31]. Lan Shaoyang [45] investigated the mechanism by observing the effect of YCHT on the expression levels of hepatic NTCP in rats in cholestasis and damp-heat syndrome models. A study has proved that YCHT ameliorates concanavalin A-induced hepatitis through its inhibitory action against the production of inflammatory cytokine and its intensive action on the production of anti-inflammatory cytokines [46]. Tzung-Yan Lee stated that YCHT can alleviate hepatic oxidative stress and inhibit fatty acid synthesis in obese mice with steatosis. This finding supports that YCHT contributes to the reduction of serum triglyceride and unsaturated fatty acid concentrations [26], which is consistent with the results of this study. The pretreatment of YCHT decreased CPZ-induced elevation in the serum levels of ALT, AST, TBIL, TC, TG, and LDL-C and up-regulated ALB. Histological examination revealed the suppression of liver injury.

Overall, the combination of LC/GC–MS-based metabolic analysis, animal modeling, and biochemical analysis in this study enabled the characterization of the cholestatic liver injury induced by CPZ in BA and FFA metabolism. This study is the first to use the combination of BAs and FFAs in serum to characterize cholestasis liver injury and evaluate the protective effect of YCHT. The data in this study indicated that YCHT may serve as protective agent against cholestasis liver injury induced by CPZ in rats. However, evidences for elucidating the mechanism on the protective effect of YCHT against this injury is still lacking. Therefore, further research should focus on the transporters of BAs and FFAs to provide the potential interpretation of the hepatoprotective effects of YCHT.

## Conclusions

The results in this study indicated that YCHT exerted a protective effect on the cholestatic liver injury induced by CPZ. The variance of BA and FFA concentrations can be used to evaluate the cholestatic liver injury caused by CPZ and the hepatoprotective effect of YCHT.
